# Green Synthesis of Silymarin–Chitosan Nanoparticles as a New Nano Formulation with Enhanced Anti-Fibrotic Effects against Liver Fibrosis

**DOI:** 10.3390/ijms23105420

**Published:** 2022-05-12

**Authors:** Abdullah Saad Abdullah, Ibrahim El Tantawy El Sayed, Abdel Moneim A. El-Torgoman, Abul Kalam, S. Wageh, Maher A. Kamel

**Affiliations:** 1 Department of Chemistry, Faculty of Science, Menoufia University, Shebin El Koom 13829, Egypt; abdullah.saad82@science.menofia.edu.eg (A.S.A.); abdelmoneam.abdelkader@science.menofia.ed (A.M.A.E.-T.); 2 Department of Chemistry, Faculty of Education, Thamar University, Thamar 63022, Yemen; 3 Research Center for Advanced Materials Science (RCAMS), King Khalid University, P.O. Box 9004, Abha 61413, Saudi Arabia; abulkalam@kku.edu.sa; 4 Department of Chemistry, College of Science, King Khalid University, P.O. Box 9004, Abha 61413, Saudi Arabia; 5 Department of Physics, Faculty of Science, King Abdulaziz University, Jeddah 21589, Saudi Arabia; wswelm@kau.edu.sa; 6 Physics and Engineering Mathematics Department, Faculty of Electronic Engineering, Menoufia University, Menoufia 32952, Egypt; 7 Department of Biochemistry, Medical Research Institute, Alexandria University, Alexandria 21516, Egypt

**Keywords:** liver fibrosis, silymarin, chitosan nanoparticles, epigenetics, microRNAs, oxidative stress

## Abstract

Background: Silymarin (SIL) has long been utilized to treat a variety of liver illnesses, but due to its poor water solubility and low membrane permeability, it has a low oral bioavailability, limiting its therapeutic potential. Aim: Design and evaluate hepatic-targeted delivery of safe biocompatible formulated SIL-loaded chitosan nanoparticles (SCNPs) to enhance SIL’s anti-fibrotic effectiveness in rats with CCl_4_-induced liver fibrosis. Methods: The SCNPs and chitosan nanoparticles (CNPs) were prepared by ionotropic gelation technique and are characterized by physicochemical parameters such as particle size, morphology, zeta potential, and in vitro release studies. The therapeutic efficacy of successfully formulated SCNPs and CNPs were subjected to in vivo evaluation studies. Rats were daily administered SIL, SCNPs, and CNPs orally for 30 days. Results: The in vivo study revealed that the synthesized SCNPs demonstrated a significant antifibrotic therapeutic action against CCl_4_-induced hepatic injury in rats when compared to treated groups of SIL and CNPs. SCNP-treated rats had a healthy body weight, with normal values for liver weight and liver index, as well as significant improvements in liver functions, inflammatory indicators, antioxidant pathway activation, and lipid peroxidation reduction. The antifibrotic activities of SCNPs were mediated by suppressing the expression of the main fibrosis mediators TGFβR1, COL3A1, and TGFβR2 by boosting the hepatic expression of protective miRNAs; miR-22, miR-29c, and miR-219a, respectively. The anti-fibrotic effects of SCNPs were supported by histopathology and immunohistochemistry (IHC) study. Conclusions: According to the above results, SCNPs might be the best suitable carrier to target liver cells in the treatment of liver fibrosis.

## 1. Introduction

Chronic liver disease (CLD) is a principal cause of death globally, encompassing a wide spectrum of disorders with varying or unknown etiologies. In 2017, an estimated 1.32 million people died worldwide directly due to liver cirrhosis complications [1].

Akçalı defines hepatic fibrosis as a dynamic pathophysiological process characterized by dysplasia of intrahepatic connective tissue that occurs as a result of sustained and chronic liver injury caused by a variety of pathogenic factors [2], including viral hepatitis, alcohol consumption, nonalcoholic fatty liver disease (NAFLD), nonalcoholic steatohepatitis (NASH), and toxic chemicals such as carbon tetrachloride (CCl_4_). Over time, liver fibrosis may develop into cirrhosis and cancer of the liver, which are both fatal in the late stages [3]. During hepatic fibrogenesis, extracellular matrix (ECM) proteins such as collagen, fibronectin, and matricellular proteins are excessively generated by activated hepatic stellate cells (HSCs), which cause structural and functional distortions in the liver and are thus a major feature of a cirrhotic liver [4]. In response to chronic inflammation, quiescent HSCs gradually differentiate into myofibroblast-like cells, which are distinguished by the appearance of cytoskeleton protein smooth muscle actin (α-SMA) and collagen, which are regarded to be indicators for HSC activation. Among the most powerful activators of HSCs, transforming growth factor-beta 1 (TGFβ-1) is the one that converts them from static HSCs to the phenotypic of myofibroblasts, which express α-SMA [5,6]. Additionally, an interrelationship between the nuclear factor erythroid-2-related factor 2 (Nrf2) and TGFβ-1 pathways has been reported to promote the development of HCC [7]. Previous studies have reported that Nrf2 inhibits antagonistically fibrotic TGFβ-1 signaling and that TGFβ-1 enhances the formation of reactive oxygen species (ROS) via blocking Nrf2 [8].

MicroRNAs (miRNAs) are short non-coding RNAs (20–22 nucleotides) that operate as posttranscriptional regulators of gene expression and play a crucial role in controlling hepatic fibrogenesis by regulating the expression of numerous signaling components, transcription factors, and cofactors [9]. Many members of the miRNA family have pro- or anti-fibrotic functions by targeting genes individually or collectively through particular signaling pathways to activate HSCs and contribute to liver fibrosis [10]. MiRNAs act by binding to their targets’ incomplete complementary sequences at the 3′ untranslated region (3′ UTR) and causing mRNA degradation or repression [11]. Several key players of liver fibrosis have been demonstrated to be targets of different miRNAs including; miR-22 [12], miR-29c [13], and miR-219a [14] during liver fibrosis. TGFβR1 [15], COI3A1 [16], and TGFβR2 genes are three of the genes that have been suggested as potential targets for miRNAs and are predominantly implicated in the fibrogenesis process [17].

Silymarin (SIL) is an important hepatoprotective complex derived from the seeds of milk thistle (*Silybum marianum* (L.) Gaertn). Despite the hepatoprotective action of SIL, poor bioavailability is the main downside of SIL oral administration [18]. The low bioavailability of SIL may be a result of its poor water solubility in gastric pH, poor permeability through the epithelial cells in the gut, and rapid elimination [19]. To overcome this obstacle, several formulations of SIL have been developed to increase the bioavailability of SIL. The nanotechnology approach may play a major role in improving the bioavailability and therapeutic properties of SIL [20]. In our previous work, we successfully synthesized SIL-loaded gold nanoparticles with high entrapment efficiency and loading capacity, and the in vivo study confirmed enhanced anti-fibrotic activities [17].

To surpass the gastrointestinal obstacles of SIL bioavailability, chitosan (Ch) was chosen in the present study. Ch is a natural origin-based polymer with versatile functional groups, mucoadhesive capabilities, the permeating-enhancing effect, controlled drug release, and efflux inhibition [21]. All of these properties make Ch a potential candidate that meets the ideal properties for an effective SIL delivery system [22]. Chitosan nanoparticles (CNPs) have been reported to have greater immune-boosting, anticancer, and antimicrobial properties than those of Ch [23]. In the present study, we aimed to synthesize and characterize silymarin–chitosan nanoparticles (SCNPs) for efficient and selective delivery of silymarin to target liver cells. We investigated the potential of SCNPs to improve the oral delivery and bioavailability of silymarin, as well as the anti-fibrotic efficacy of SCNPs in a rat model of CCl_4_-induced liver fibrosis compared to native SIL.

## 2. Results

### 2.1. Characterization of Nanoparticles

#### 2.1.1. TEM Analysis of CNPs

**Figure 1:**
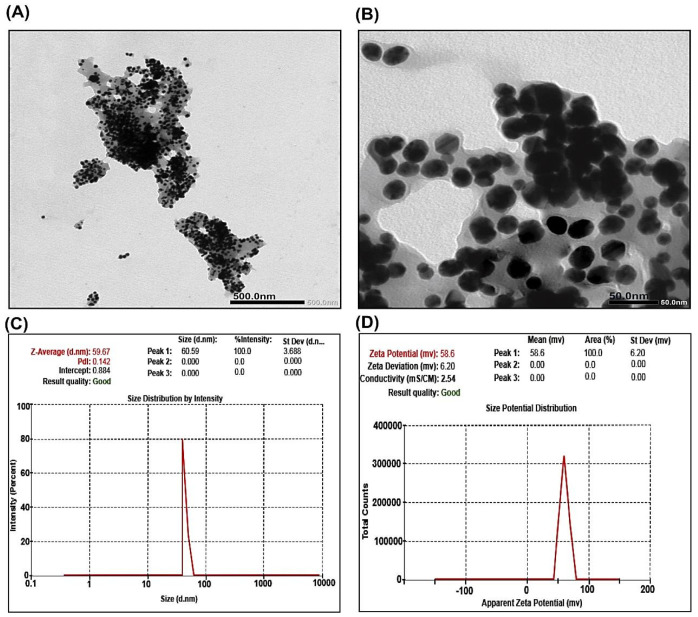
Transmission electron microscopy images of CNPs: (**A**,**B**) average particle size around 50 ± 5 nm; (**C**) particle size distribution of CNPs assessed by dynamic light scattering (DLS) at 59.67 nm; (**D**) zeta potential distribution of CNPs by dynamic light scattering (DLS) shows positive charge at +58.6 mV.

The characterization of CNPs using TEM investigation showed the spherical shape of CNPs. As shown in the TEM images, CNPs have a spherical uniform solid and opaque dense structure and have an almost uniform distribution of the particle size. However, CNPs of cross-linking (5%) tended to be low aggregates. The average particle size of CNPs was around 50 ± 5 nm as shown in Figure 1A,B.

#### 2.1.2. DLS and Zeta Potential Measurements of CNPs

Hydrodynamic dimensions and surface charge have an important role in the characterization of particles, which were measured using DLS and Zeta Potential. They give the NP dispersion, size, and charge. CNP size, as shown in Figure 1C, was found at 59.67 nm. For the polydispersity of CNPs, polydispersion index (PDI) values were estimated at around 0.142. The results clearly demonstrated that the zeta potential of the synthesized CNPs was observed at +58.6 mV (Figure 1D).

#### 2.1.3. TEM Analysis of SCNPs

**Figure 2:**
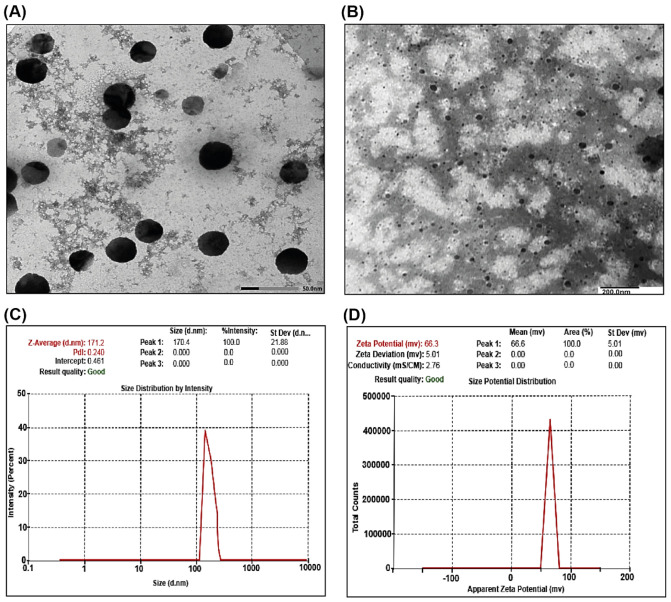
Transmission electron microscopy images of SCNPs: (**A**,**B**) average particle size around 78 ± 6 nm; (**C**) particle size distribution for synthesized SCNPs by dynamic light scattering shows the average size at 170.4 nm; (**D**) zeta potential of SCNPs shows stable positive charge at +66.3 mV.

TEM assessments were performed to validate the size and shape of the SCNPs. Figure 2A,B shows TEM images of SCNPs, which indicated that the NPs are disseminated as separate NPs with clear-cut spherical in the shape and homogeneously dispersed with average particle size around 78 ± 6 nm.

#### 2.1.4. DLS and Zeta Potential Measurements of SCNPs

Distribution curves of particle size revealed only one peak, as well as a comparatively low Polydispersity index. The particle size of SCNPs was shown to be 170.4 nm (Figure 2C). The results showed that the investigated sample of SCNPs presented highly positively charged values at +66.3 mV, indicating that the synthesized NPs displayed increased dynamic stability as shown in Figure 2D.

#### 2.1.5. FT-IR Study

**Figure 3:**
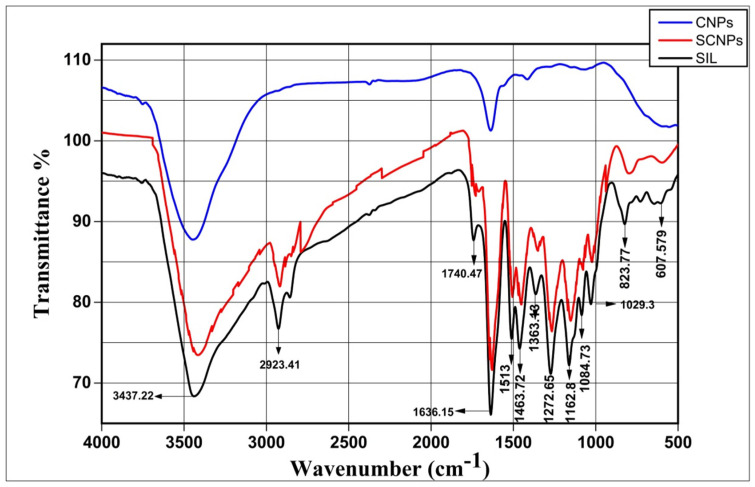
FT-IR spectra of SIL, CNPs, and SCNPs.

FTIR data show the percentage of transmission spectral to identify any potential chemical reaction that might have occurred between the drug and the polymer nanoparticle. In addition, it is necessary to define functional groups. Figure 3 shows the FT-IR spectra of pure drug SIL, CNPs, and SCNPs. Due to the presence of different functional groups, the FT-IR spectra showed characteristic bands. The FT-IR spectrum of pure drug SIL showed an absorption band at 3437.22 cm^−1^ (O–H stretching, Phenols/Alcohols), 2923.41 cm^−1^ (C–H stretching, Alkyl), 1740.47 cm^−1^ (C=O stretching, Esters), 1636.15 cm^−1^ (C=O stretching), 1513–1463.72 cm^−1^ (skeleton vibration of aromatic C=C ring stretching), 1363.13 cm^−1^ (N-O stretching, Nitro compound), 1272.65 cm^−1^ (C–O stretching, Carboxyl acid),1162.8 cm^−1^ (C–O stretching, Esters), 1084.73 cm^−1^ (benzopyran ring vibrations), 1029.3 cm^−1^ (C–O group stretching, Sulfoxide), 823.77 cm^−1^ (C-H bending, Alkenes), and 607.579 cm^−1^ (C–I stretching, halo compound). The enhanced batch of SCNPs exhibited all characteristic peaks and matched with individual vibrational bands of pure drug SIL with a slight shift in peak positions. As a result, the drug showed no chemical interactions with the excipients in the formulation.

#### 2.1.6. Drug Entrapment Efficiency and Loading Capacity of SIL in SCNPs

Entrapment efficiency (EE %) and drug loading capacity (LC %) are considered to be key parameters for the investigation of nanoparticle properties. EE % of SIL in SCNPs was indirectly measured by determining SIL content in supernatant, and the results revealed that more than 97.16 ± 2.35% of SIL was entrapped in polymer content. Conversely, LC %value was 14.27 ± 1.50%.

#### 2.1.7. In Vitro Drug Release Study

**Figure 4:**
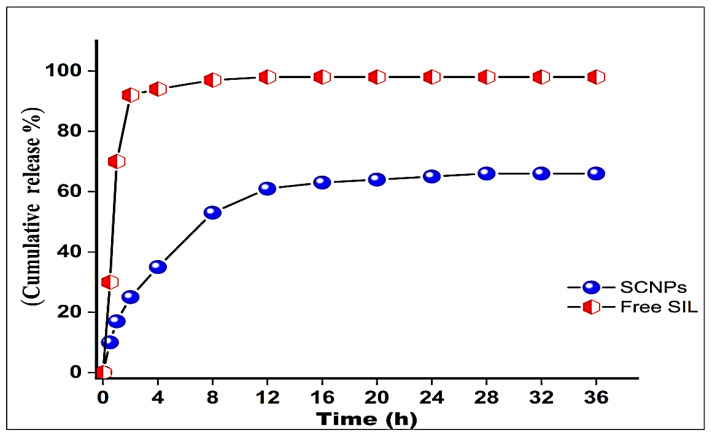
Cumulative in vitro release profile of free SIL from SCNPs. Each data point is represented as mean ± SD (*n* = 3).

The release profile of SIL from the nanoparticles was studied in vitro using PBS (pH 7.4) at 37 ± 0.5 °C, and the accumulated amount of SIL released percentage (%) at specific different time intervals was plotted versus time (h), according to the data collected from the release studies. As shown in Figure 4, a typical two-phase release pattern was shown in vitro. The first phase release pattern exhibited a relatively rapid a burst and release at an early particular point in time (~35% of the entrapped Silymarin release in 4 h) followed by the sustained and gradual release phase over an extended period of time. Approximately 66% of the drug was released around 36 h, while 92% of drug was released from pure silymarin during the first 2 h. The detected initial rapid release may be due to the dispersion of the drug present on the surface of SCNPs, accompanied by a slower prolonged release of the SCNPs.

### 2.2. In Vivo Studies

#### 2.2.1. Body Weight Gain, Liver Weight, and Liver Index (%)

**Figure 5:**
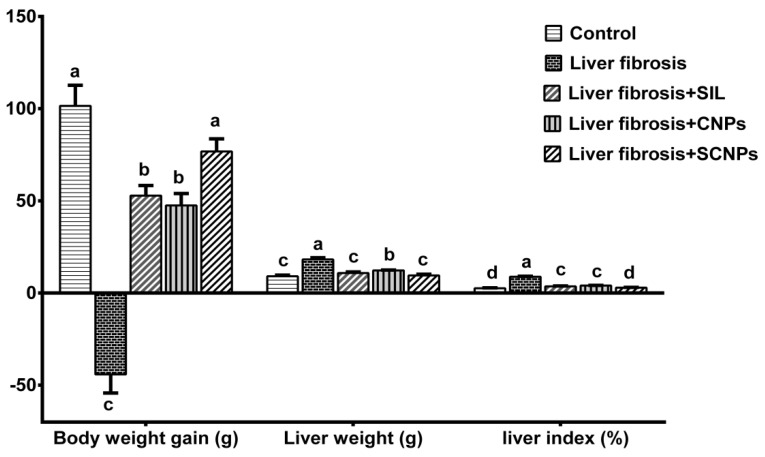
Effect of different groups on body weight gain, liver weight, and liver index (%). Data are presented as mean ± SD (*n* = 6). The means of the groups in the same column with the same superscript letter are not significantly different, while the means with different letters are significantly differed according to the ANOVA test, followed by the post hoc test (Tukey). At *p* ≤ 0.05, the difference was statistically significant.

In comparison to the control group, CCl_4_ exposure significantly lowered body weight gain as well as increased liver weight and liver index. CCL_4_-intoxicated rats treated with any of the treatments gained significantly more body weight and had lower liver weight and liver index values than the CCl_4_-untreated group, with the best effect seen in rats treated with SCNPs, which showed completely normal values (Figure 5).

#### 2.2.2. Serum Liver Function Markers

CCl_4_-rats show considerably higher serum activities of AST, ALT, and ALP as well as bilirubin levels and significantly lower albumin levels as compared to control rats. CCl_4_-rats treated with SIL, CNPs, or SCNPs show substantial improvements in all liver serum indicators compared to untreated rats. The best effects were shown in CCl_4_-rats treated with SCNPs, which revealed no significant differences when compared to control rats (Table 1).

#### 2.2.3. Hepatic Redox Parameters and TGFβ-1

**Figure 6:**
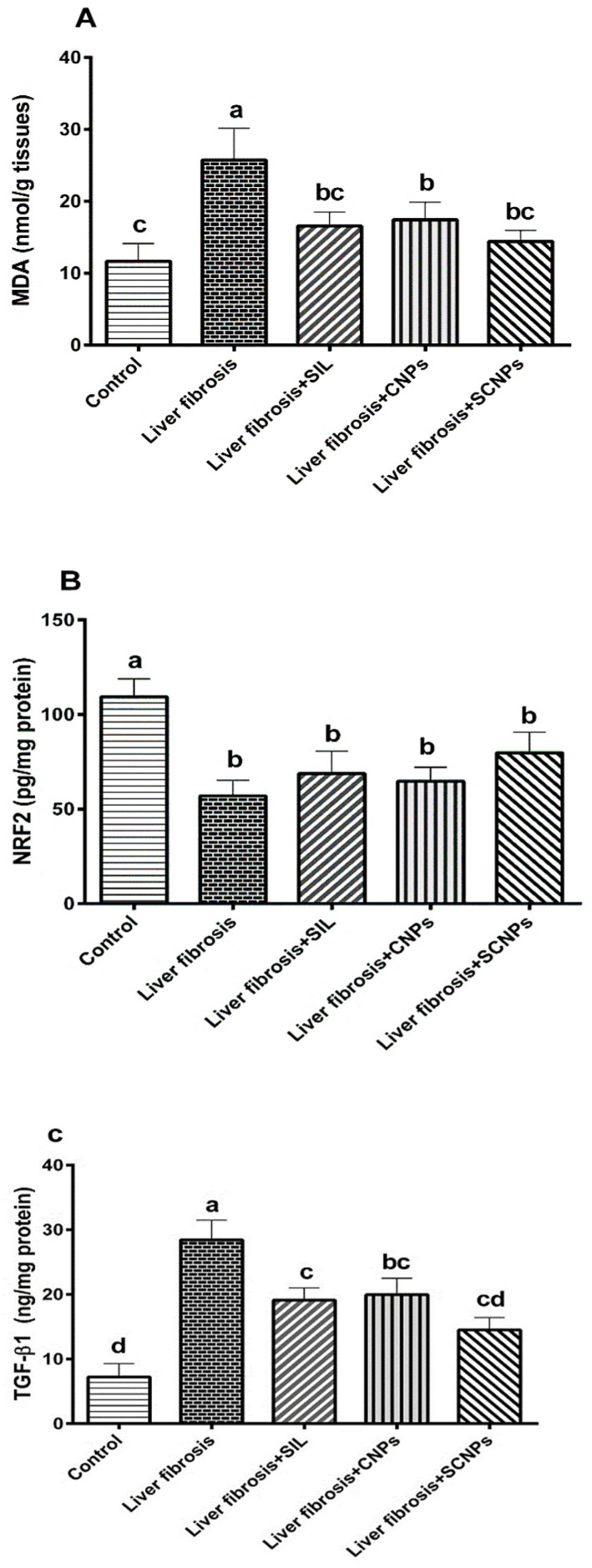
Effect of different groups on (**A**) MDA; (**B**) NRF2; (**C**) TGF-β levels in hepatic tissue homogenate. Data are presented as mean ± SD (*n* = 6). The means of the groups in the same column with the same superscript letter are not significantly different, while the means with different letters are significantly differed according to the ANOVA test, followed by the post hoc test (Tukey). At *p* ≤ 0.05, the difference was statistically significant. Abbreviations: MDA, malondialdehyde; NRF2, nuclear factor-erythroid 2-related factor 2; TGF-β, transforming growth factor-β.

The hepatic content of malondialdehyde (MDA), Nrf2, and TGF- is shown in Figure 6A–C. Compared to control rats, CCl_4_-rats showed markedly increased hepatic MDA and TGF- levels. When CCl_4_-rats were treated with SIL, CNPs, or SCNPs, the hepatic content of MDA and TGF- was significantly reduced than in untreated rats, with the highest effect shown in the rats treated with SCNP, which outperformed SIL and CNPs in terms of response. In contrast, when CCl_4_-treated rats were compared to control rats, where the level of nuclear Nrf2 was significantly lower. The hepatic content of Nrf2 in CCl_4_-rats was moderately affected by CNPs and SIL treatment. The Nrf2 level in the CCl_4_-rats treated with SCNPs increased significantly compared to the untreated rats, although it was still lower than the control value.

### 2.3. Molecular Analysis

#### 2.3.1. MicroRNAs Expression

**Figure 7:**
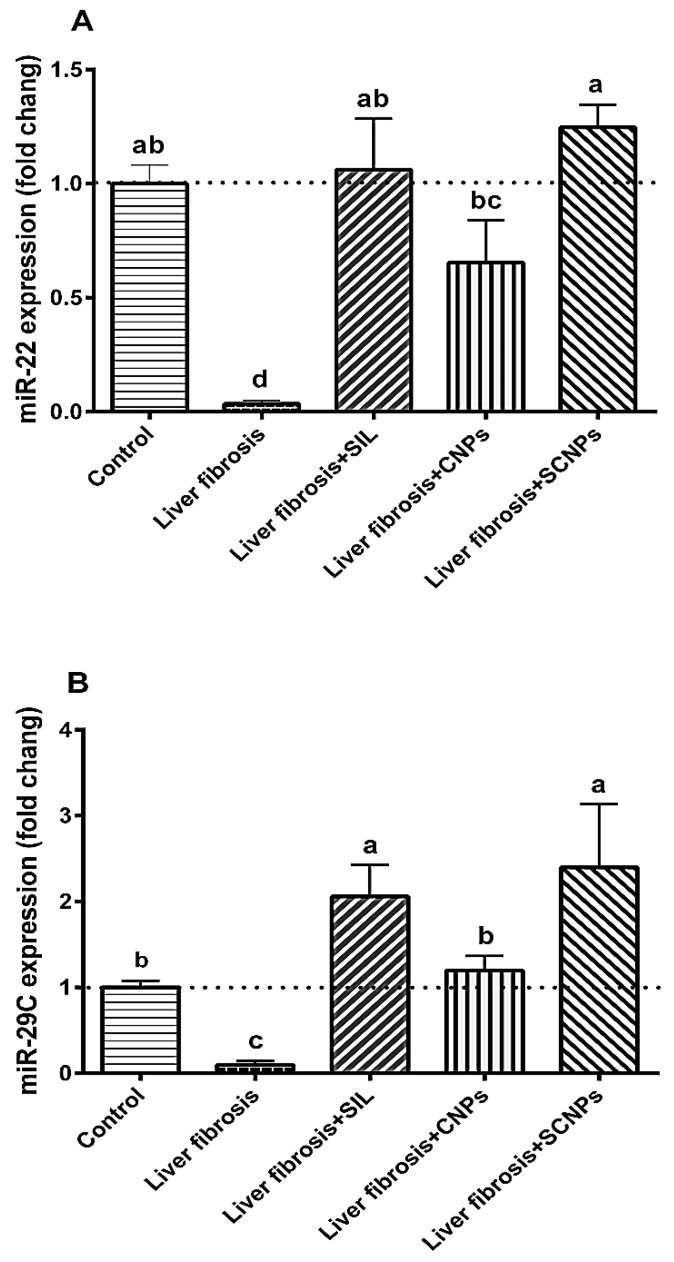

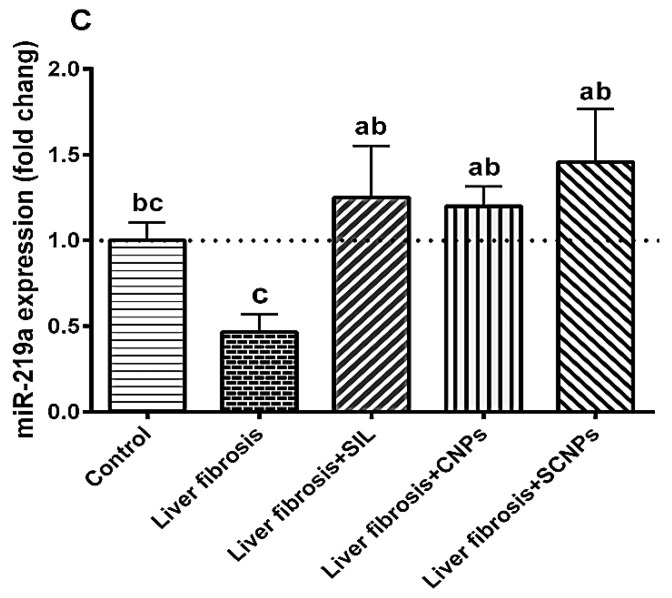
Hepatic expressions of miRNAs: (**A**) miR-22; (**B**) miR-29c; and (**C**) miR-219a. Data are presented as mean ± SD (*n* = 6). The means of the groups in the same column with the same superscript letter are not significantly different, while the means with different letters are significantly differed according to the ANOVA test, followed by the post hoc test (Tukey). At *p* ≤ 0.05, the difference was statistically significant.

In this study, the results of miR-22 expression in hepatic are shown in Figure 7A. Hepatic miR-22 expression was severely suppressed in CCl_4_-intoxicated rats, which was around 3.5% of that in the control group. The hepatic tissue was significantly preserved against CCl_4_-induced suppression of miR-22 when all of the treatments were used in the current study. The best therapeutic impact was marked in the group treated with SCNPs followed by SIL and CNPs.

Intoxicated rats showed a significantly suppressed hepatic miR-29c expression (approximately 9.6% of the control level). The expression of miR-29c was significantly upregulated in the SIL and CNP-treated CCl_4_-intoxicated rats compared to the untreated rats. The expression levels of miR-29c in rats injected with CCl_4_ and orally administered with SCNPs were significantly higher than the control values (Figure 7B).

In the CCl_4_-rats, miR-219a expression was significantly downregulated than in the control group. MiR-219a expression was significantly normalized in CCl_4_-intoxicated rats treated with any of the present regimens, with the most obvious effect shown in rats treated with SCNPs, which showed even higher expression levels than control rats (Figure 7C).

#### 2.3.2. The Expression of the Target Genes

**Figure 8:**
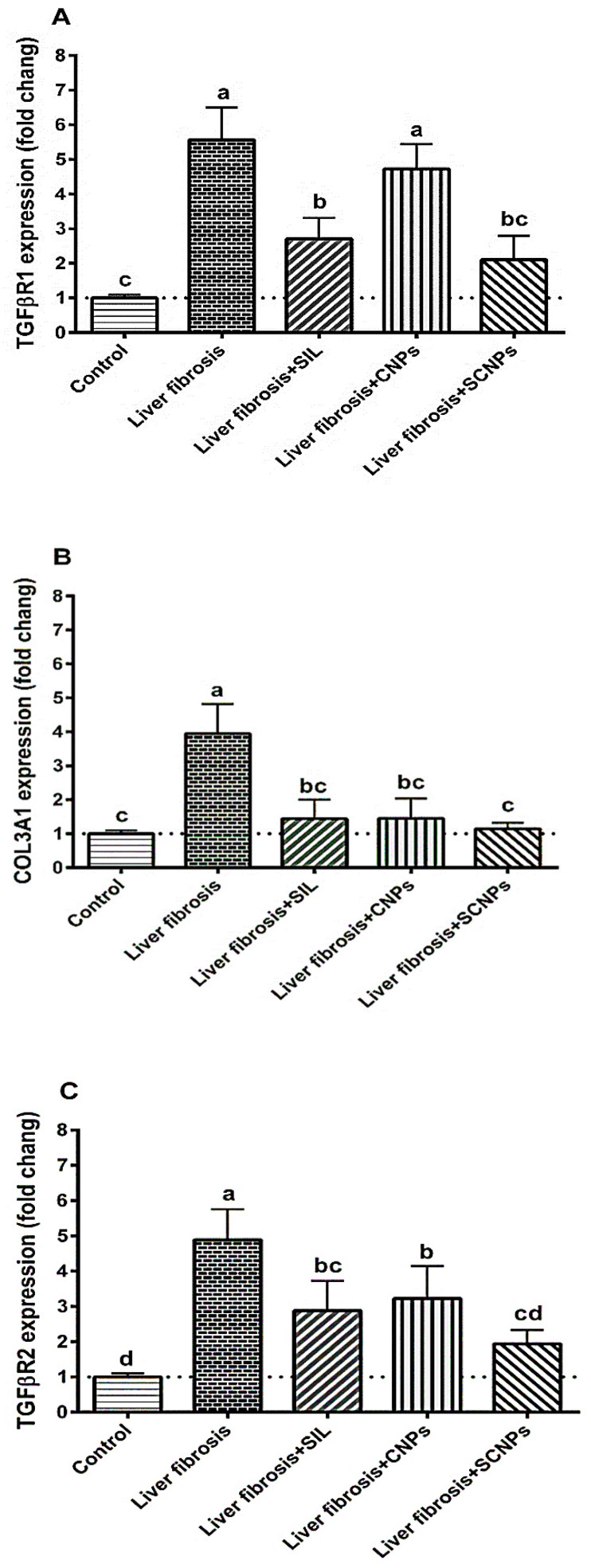
Hepatic expression of target genes: (**A**) TGFβR1; (**B**) COL3A1; and (**C**) TGFβR2. Data are presented as mean ± SD (*n* = 6). The means of the groups in the same column with the same superscript letter are not significantly different, while the means with different letters are significantly differed according to the ANOVA test, followed by the post hoc test (Tukey). At *p* ≤ 0.05, the difference was statistically significant. Abbreviations: TGFβR1, transforming growth factor-beta receptor I; COL3A1, collagen type III alpha 1; TGFβR2, transforming growth factor-beta receptor II.

TGFβR1 expression was remarkably upregulated in CCl_4_-rats compared with control rats. CNP treatment on CCl_4_-intoxicated rats had little effect, whereas SIL treatment dramatically downregulated TGFβR1 hepatic expression in CCl_4_-intoxicated rats compared to untreated rats. The best ameliorative effects presented in SCNP-treated rats, which had their expression levels normalized (Figure 8A).

The CCl_4_-induced liver-damaged rats showed significant induction of COL3A1 hepatic expression compared with the control rats. The expression of COL3A1 was significantly suppressed in CCl_4_-rats treated with SIL or CNPs compared to untreated rats, whereas the expression was significantly and completely normalized in SCNPs-treated rats (Figure 8B).

Compared with the control group, CCl_4_-intoxicated rats had dramatically enhanced TGFβR2 hepatic expression. CNP treatment had little influence on the expression of TGFβR2 in CCl_4_-rats, whereas SIL treatment dramatically reduced TGFβR2 expression compared to untreated rats. The expression levels of TGFβR2 in CCl_4_-rats administered with SCNPs were almost normal. (Figure 8C).

#### 2.3.3. Correlation Studies

**Figure 9:**
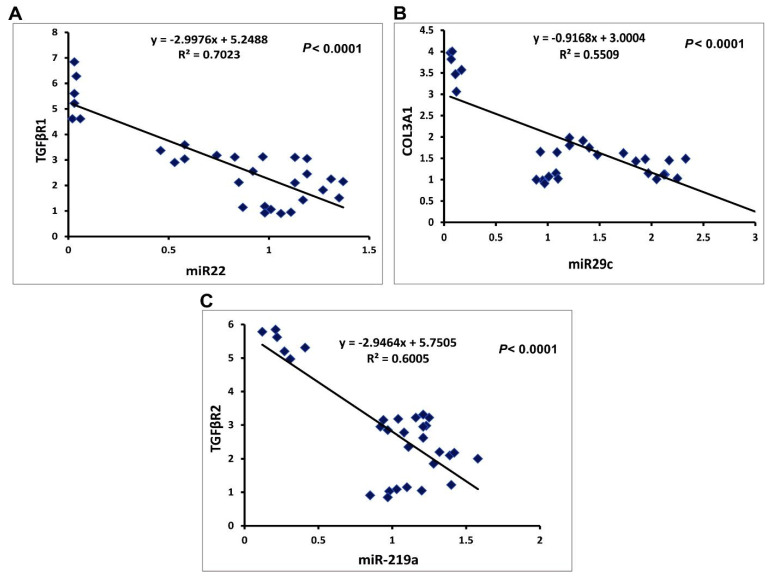
The Pearson significant correlations between miRNA genes and their target genes in the hepatic of rats treated with different study groups: (**A**) correlation curve between miR-22 and TGFβR1 expression; (**B**) correlation curve between miR-29c and COL3A1expression; (**C**) correlation curve between miR-219a and TGFβR2 expression.

The expression levels of miRNAs were adversely correlated with their targets, according to a statistical analysis using Pearson correlation. TGFβR1 is inversely correlated with miR-22 (Figure 9A), COL3A1 is inversely correlated with miR-29c (Figure 9B), and TGFβR2is inversely correlated with miR-219a (Figure 9C).

### 2.4. Histopathological Analysis

#### 2.4.1. Liver Morphology

**Figure 10:**
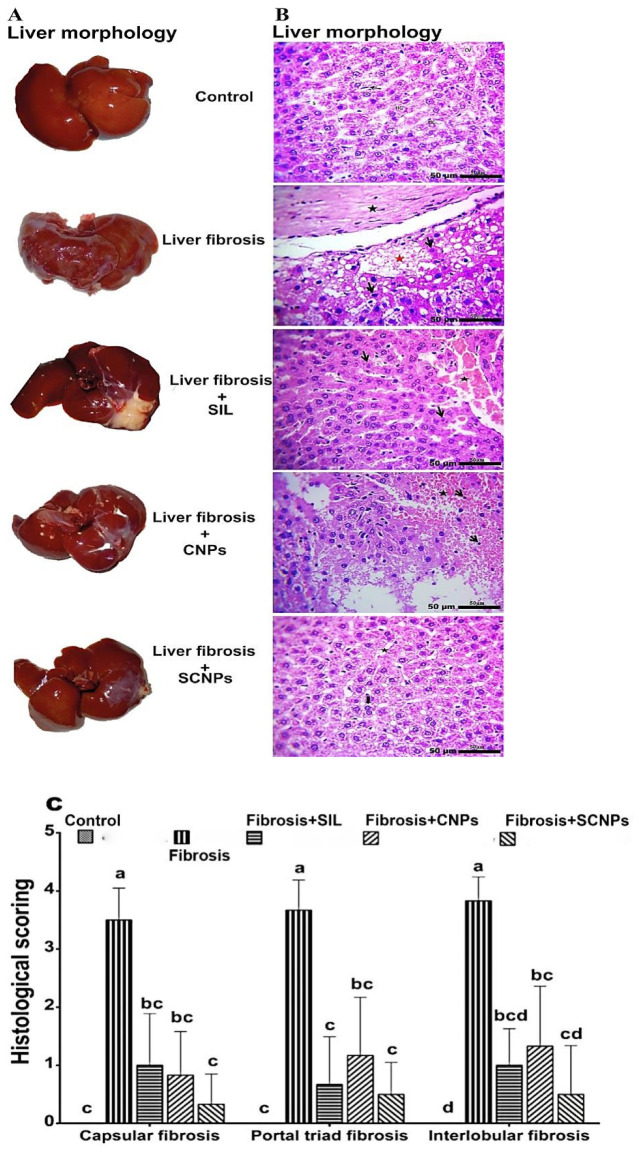
(**A**) Photographs of the liver after tissue was harvested from the animals at the end of the experiment; (**B**) liver sections stained with H&E under the microscope: (Control) showing normal hepatocytes (HC), central vein (CV), sinusoids (S), and kupffer cells (arrow). (Fibrosis) showing mature collagen (star) subscapular necrosis replaced by edema (red star) followed by pyknotic nuclei (arrow). (Fibrosis + SIL) showing mild to moderate pyknotic hepatocytes (arrows) congested and widening sinusoids (star). (Fibrosis + CNPs) showing hepatic necrosis (star) replaced by debris, erythrocytes and a few lymphocytes (arrow). (Fibrosis + SCNPs) showing acute cell swelling of hepatocytes with mild pyknotic in a few hepatocytes (arrow); (**C**) histologic grading of H&E-stained sections for interlobular fibrosis, portal triad fibrosis, and capsular fibrosis, as essential and associated lesions markers for liver fibrosis among treatment groups was performed. Data are presented as mean ± SD (*n* = 6). The means of the groups in the same column with the same superscript letter are not significantly different while the means with different letters are significantly differed according to the ANOVA test, followed by the post hoc test (Tukey). At *p* ≤ 0.05, the difference was statistically significant.

Figure 10A shows images of gross examination of the liver after excision from animals. The olive oil-treated control group showed a healthy liver with a soft, smooth, and shiny surface character. Nevertheless, the liver of CCl_4_-intoxicated rats exhibited a nodular appearance with a rough, stiff, hard spotty surface and no luster; in addition, the faint red color indicated the flow of blood through the liver decreased, resembling human liver fibrosis. After treatment, mild anomalies in liver morphology were observed in the rats treated with CNPs. The livers of SIL-treated rats were healthier than those of CCL_4_-untreated rats, with fewer nodular abnormalities and a smoother surface. SCNP treatment significantly inhibited CCl_4_-induced hepatic fibrosis, resulting in significantly improved gross appearance, a much healthier appearance, with bright, shiny, and smooth surfaces in rat livers.

#### 2.4.2. Histopathology of Liver Tissue

In the livers of control rats, all hepatic architectures, along with the central vein and hepatic cords, hepatocytes, sinusoids, and the portal triad, were normal. The CCl_4_-rat liver sections exhibited a considerable enlargement of the hepatic vein and congested blood vessels, as well as pronounced portal fibrosis with mature collagen fibrils deposited and fibroblast hyperplasia. The treated groups exhibited varying levels of the preceding change’s ameliorative effects. The SIL-treated rats improved somewhat, showing mild to moderate pyknotic hepatocytes congested and widening sinusoids, while the liver sections of CNPs treated rats showed focal necrosis, which was replaced by necrotic debris and extravasated erythrocytes. The liver sections of the rats that received SCNPs showed apparently normal hepatic architectures, with diplocytes of the few hepatocytes, and a slight widening of sinusoids (Figure 10B).

#### 2.4.3. Histological Grading of Fibrosis

The degree of histological alterations in the liver (interlobular fibrosis, portal triad fibrosis, and capsular fibrosis) from 0 to 4+ grades was reported as the index for liver fibrosis among the studied groups (Figure 10C).

#### 2.4.4. Masson’s Trichrome (MT) Staining of Liver Tissue

**Figure 11:**
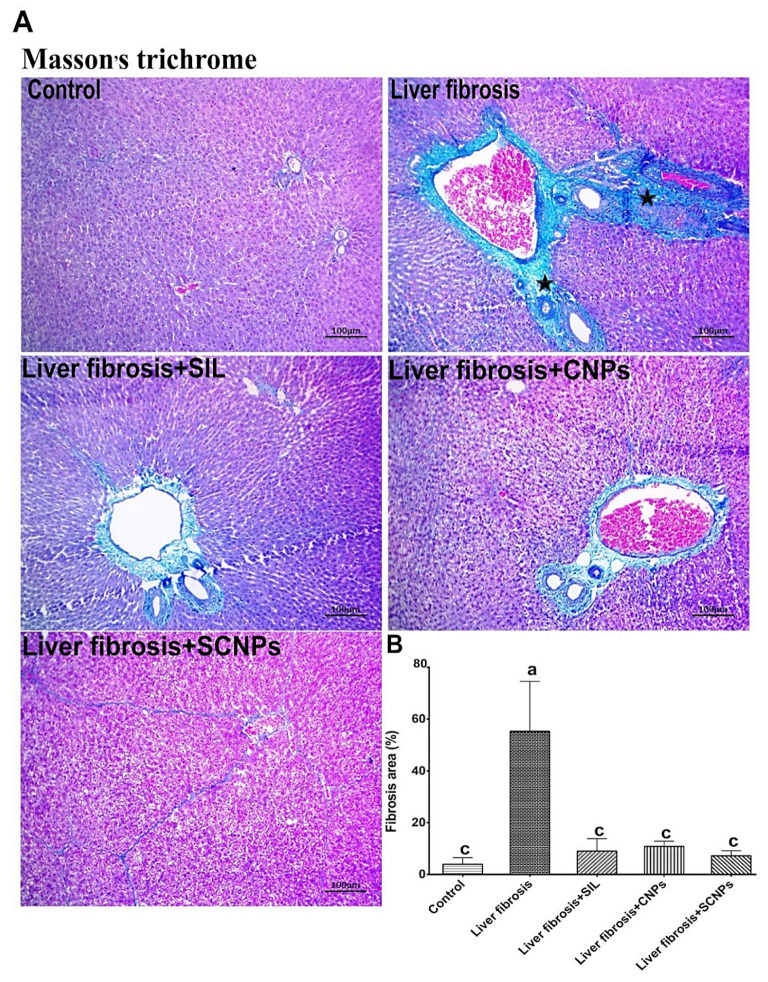
(**A**) Mason’s trichrome (MT) stained liver sections under microscope; CCl_4_-induced hepatic fibrosis shows intense blue satiable materials in the portal area, which extended to the interlobular (stars); however, other groups show characters of fibrosis unevenly except SCNPs, which show a significantly reduced degree of liver fibrosis. (**B**) Fibrosis scores of the different groups were determined by ImageJ software as MT areas percent (fibrosis area % per 10 fields in magnification power ×100). Data are presented as mean ± SD (*n* = 6). The means of the groups in the same column with the same superscript letter are not significantly different, while the means with different letters are significantly differed according to the ANOVA test, followed by the post hoc test (Tukey). At *p* ≤ 0.05, the difference was statistically significant.

In order to identify the collagen fibers in the liver tissue for the evaluation of fibrosis and its recovery, Masson’s trichrome staining was used. The results of the MT staining demonstrating accumulation of matured collagen fibers (stained blue) during CCl_4_-induced hepatic fibrosis, and the role of treatment groups to prevent collagen synthesis and deposition in the liver are depicted in Figure 11A,B. In the control group, MT staining of the normal liver revealed no collagen deposition. Liver sections stained with MT stain reflected changes in the liver architecture and showed accumulation of matured collagen fibers (stained blue), and prominent fibrosis in sections prepared from the group of CCl_4_-induced hepatic fibrosis in rats compared to normal control, whereas the liver sections from CCl_4_-intoxicated rats administered by SIL and CNPs had a decrease in these changes, with fewer fibers as compared with CCl_4_-induced group. As compared to SIL and NPs as monotherapy, the highest results were shown in rats treated with SCNPs, which revealed apparently normal hepatic architecture, considerably decreased degree of liver fibrosis, and ameliorated CCl_4_-induced hepatic fibrosis. As a result, the treatment efficacy of drug-loaded nanoparticles against chronic hepatic fibrosis may be determined directly by MT staining of extracellular matrix (ECM) deposition in hepatic tissue.

#### 2.4.5. Immunohistochemistry (IHC) of αSMA

**Figure 12:**
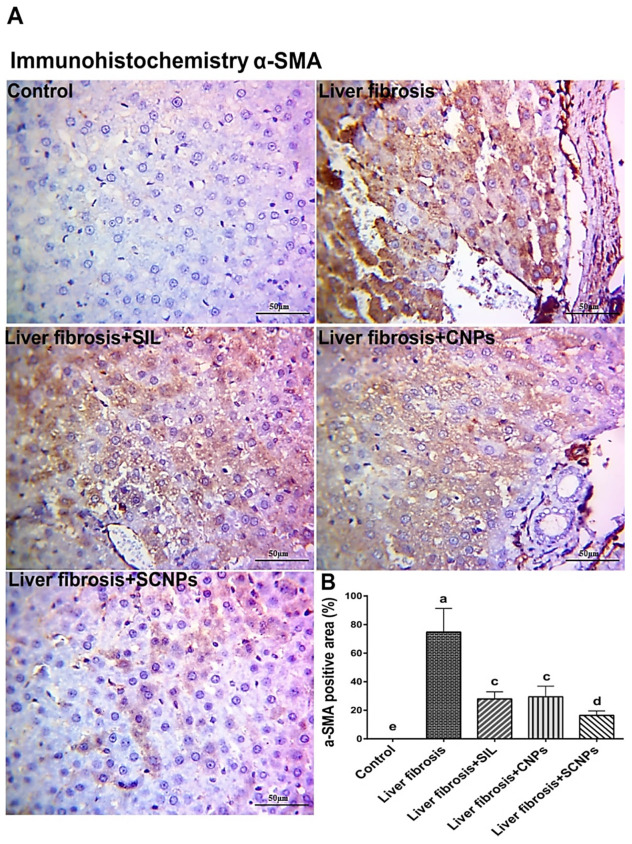
(**A**) α-SMA immunohistochemistry (IHC) staining in liver sections. The liver section of the CCl_4_ rats showed high immunoreactivity; nevertheless, the number of α-SMA immunoreactive cells was markedly reduced by the treated group of SIL, CNPs, and SCNPs, with the strongest result in the SCNPs group, which demonstrated a potent response in the attenuation of IHC reactions. (**B**) α-SMA immunohistochemistry reactions positive area (average number of positive cells per 10 field’s high power ×400) quantified with Image J. Data are presented as mean ± SD (*n* = 6). The means of the groups in the same column with the same superscript letter are not significantly different, while the means with different letters are significantly differed according to the ANOVA test, followed by the post hoc test (Tukey). At *p* ≤ 0.05, the difference was statistically significant.

In order to estimate activation of the hepatic stellate cell (HSC), IHC was carried out on liver tissue with an anti-αSMA (smooth muscle actin) antibody, one of the crucial indicators of the fibrogenesis rate (Figure 12A,B). Our findings revealed that in the control group, there were hardly any positive α-SMA cells and no immunohistochemistry reactions. α-SMA immunohistochemistry exhibited high immunoreactivity in the case of rats with CCl_4_-induced liver fibrosis as compared to the control group, confirming that HSCs were activated in the CCl_4_-induced liver fibrosis model. A section of a liver obtained from the CCl_4_-intoxicated group treated with SIL and CNPs showed moderate α-SMA immunoreactive cells, as compared with the CCl_4_-induced liver fibrosis group. The livers of rats that received SCNPs had a staining pattern comparable to control animals with desultory α-SMA positivity.

It was clearly shown that CCl_4_ treatment caused a more than three-fold increase in α-SMA level, which upon treatment with the different treatment groups, decreased, particularly in the nanoparticles conjugation with the drug groups, which reached comparable values to the control animals, indicating an attenuation of the fibrogenic properties of HSCs after administration of SCNPs.

## 3. Discussion

The scope of this work was to prepare and characterize silymarin (SIL)-loaded chitosan nanoparticles (SCNPs) as a new potential hepatotherapeutic agent, which was formulated by tying and loading the SIL molecule on CNPs to improve the antifibrotic effects of SIL in a rat model of CCl_4_-induced liver fibrosis.

Due to their good physicochemical characteristics being diverse, CNPs were used as a drug carrier, particularly for oral drugs such as SIL, because the oral drug delivery is impeded by poor absorption and instability of the drugs in the gastrointestinal tract (GI) and the presence of the mucus layer overlying the intestinal epithelium. Therefore, a polymeric drug delivery system such as chitosan is an ideal approach to enhance oral drug bioavailability and intestinal drug absorption [21]. Chitosan (Ch) has an amino group with a pKa of ~6.5, which becomes fully protonated at pH of ~4, resulting in an increase in the positivity of Ch [24,25]. The interaction between the positively charged CNPs and mucin (negatively charged) leads to an increase in contact time between the drug and the absorptive surface. In addition, the absorption-promoting effect of chitosan is due to its muco-adhesion properties and transient opening of the tight junctions of the mucosal cell membrane [26]. Therefore, the chitosan-loaded drug has better mucoadhesive and permeation-enhancing properties, promoting drug absorption in the proximal part of the GI tract, including the stomach and duodenum, when compared with the Ch-free group [25,27,28,29]. In addition, Ch can inhibit some transporter proteins on the membrane of intestinal epithelial cells or enterocytes, which act as efflux pumps of drugs that contribute to the mechanism of drug resistance [30].

The ionic gelation method is based on the ionic crosslinking interaction between protonated amine groups of positively charged chitosan solution [31] and anionic phosphate groups of the cross linker, negatively charged sodium tripolyphosphate (TPP) solution [32]. The ionic interaction is significantly influenced by the charge and density of both chitosan and TPP solutions [33].

The results confirmed the successful synthesis of SIL-loaded SNPs (SCNPs) as characterized using TEM, DLS, and FT-IR. The particle size of the prepared NPs plays a critical role in the targeting, absorption, and loading of the passive drug [34], which is a necessary parameter because it can directly influence the physical stabilization, the release of drugs from nanoparticles, and can affect their bio-distribution [35]. As it has been shown, NPs in the 100–200 nm range are rapidly absorbed by phagocytes of the reticuloendothelial cells and are ideally dispersed in the liver, as has been substantiated by [36]. The size of nanoparticles determines the ability of the particles to move across the blood–brain barrier and is a critical parameter for effective internalization in cancer cells [37]. The fate of the polymer nanoparticles loaded with drugs depends primarily on their physicochemical character. NPs of small particle size (<200) have recorded increased drug accumulation through improved permeability and retention effects in tumor cells [38]. In the present study, the analysis by TEM revealed a consistent distribution within the core-shell of the trapped drug. The particles of CNPs and SCNPs were found to be disseminated as separate NPs with clear-cut spherical in the shape and homogeneously dispersed with an average particle size around 50 ± 5 and 78 ± 6 nm, respectively, and drug accumulation is predicted to be effective in target cells. The particle size in the synthesized SCNPs was identical to the ones described by [39]; however, they were different in shape and size from the ones mentioned by these authors, since they were found to be almost spherical shape and particle size range was 100–150 nm. TEM investigation has shown that the synthesized SCNPs have a far smaller particle size than those observed by dynamic light scattering (DLS). DLS measurement is dependent on hydrodynamic particle diameter and helps to give an intensity-weighted average particle size, whereas microscopic measurement techniques are based on dry particle diameter and provide the average particle size measurement [40]. DLS and zeta potential measurements have become common as simple, fast, and reproducible methods for determining the size of the particles and their surface charge [41]. Mean particle diameters of CNPs and SCNPs were equal to 59.67 and 170.04 nm, respectively. Optimally, the particle size range for a stable system should be <200 nm for nanoparticle drug delivery systems [42]. The Polydispersity Index (PDI) for CNP and SCNPs, 0.142, 0.441, respectively, represents monodispersity and ideal dispersion without aggregation of the nanoparticles. The results found that zeta potential values for CNP and SCNPs showed highly positive charged values of +58.6 and +66.3 mV, respectively, indicating that high dynamic stability was exhibited in all the systems examined. This is in conformity with Liang and his colleagues [43], who reported that positively charged nanoparticles can enter into cells by endocytosis because the nanoparticles bind tightly to the cellular membrane, which is charged negatively, and then lead to successful intracellular trafficking [44]. The positive zeta potential of CNPs may be due to the presence on their surface of free and unconjugated amine groups. Overall, high zeta potential values reflect the high physical stability of nanoparticles [39].

In the three components, CNPs, pure drug SIL, and SCNPs, the FTIR experiment was used to determine the functional groups. The FT-IR spectrum of pure SIL exhibited a distinctive stretching vibration peak of the C–O bond at 1162.8 and 1272.65 cm^−1^, and for C=C peaked at 1513, 1463.72 and 1636.15 cm^−1^. The absorption bands at 1740.47 cm^−1^ ascribed to C=O were related to the ketone group, and the ones attributed to stretching vibration of aliphatic C–H groups peaked at 2923.41 cm^−1^, and finally, those related to stretching vibration of numerous O–H groups peaked at 3437.22 cm^−1^. In addition, the FTIR spectra of CNPs showed absorption bands at 3431.45 cm^−1^ assigned to the O–H bond in Ch polymeric chain, the characteristic peak at 1632.82 cm^−1^ correspond to mixed (C=O) amide and (C=C) vibrations, and a small peak at 1434.16 cm^−1^ is attributed to the symmetric aromatic ring stretching vibration (C=C ring). FT-IR measurements showed the existence of a similar silymarin spectrum in SCNPs with minor shifts in characteristic wavenumbers indicating the conjugation of the flavonoid to the CNPs. FTIR spectrum has clearly confirmed the successful entrapment of the SIL inside the NPs without any significant chemical interaction. These results were consistent with previously published SCNPs results [45].

The high EE % is due to increased drug and polymer affinity with the same solvents. The low EE % is due to the high affinity of drugs and polymers to various solvents [46]. The entrapment efficiency depends on the content of the polymer. The results have shown that more than 97.16 ± 2.35% of SIL was encapsulated at high polymer content. The increase in entrapment efficiency can be justified by the existence of an adequate concentration of polymer to encapsulate SIL in the formulation [39]. The high encapsulation efficiency is helpful, as it carries enough drugs at the target site and increases the duration of the drug’s residence.

The therapeutic effectiveness of the drug would rely primarily on the dosage and duration of its availability on the intracellular site of action. A carrier that could release the drug steadily in the intracellular compartment at the site of action would improve the curative efficacy of the drug and preserve long-term therapeutic effects [38]. These results are in agreement with the previously reported results [47].

The drug release profile in vitro studies of the synthesized formulation at (pH 7.4) exhibited a fast freed 35% of the encapsulated drug in nanocarrier in the first 4 h, which may be due to the release of the adsorbed drug molecules on the surface of nanoparticle [48]. Subsequently, the sustained release was stable at 66% of the drug entrapped in NPs during the 36 h study period, due to the sustained release behavior of formulated NPs, and probably the hydrophobic nature of SIL [37]. Furthermore, the slow release of the drug can result from electrostatic interaction between the drug and the self-assembled micelles [49]. This biphasic pathway for SCNPs release has been previously reported, noting that differences are in the vitro study period and sustained release profile [50]. These results indicated that the incorporation of SIL into carriers with higher bioavailability improves its solubility and regulates release [51]. The controlled release property of CNPs makes it an excellent delivery system for SIL [37]. The results of the dissolution demonstrated the release of the drug from the formulation after the diffusion and corrosion of the polymer. The medium perforates the Interpenetrating Polymer Network (IPN) when the formulation comes into contact with the dissolution media, contributing to polymer chain disentanglement, inducing polymer corrosion and eventual release of SIL [50]. The initial burst release of pure SIL over zero to 4 h was observed during this investigation but was not available for sustained release essentially due mainly to its heterogeneous distribution of SCNPs [52,53]. Zero-order drug release was considered to be the best designed to suit the optimized formulation [54]. Contrastingly, the release of SCNPs from the formulation was constant and continued during 36 h, which can be due to the high polymer concentration that slows the diffusion of SCNPs within polymer droplets [55].

The in vivo studies on male Sprague–Dawley rats with CCl_4_-induced hepatotoxicity as an experimental model evaluated the anti-fibrotic effects of the formulated SCNPs in comparison with SIL solution and CNPs. The model of rat liver intoxication with CCl_4_ is one of the most suitable in vivo models of liver cirrhosis and fibrosis used for the screening of therapeutic effects of many herbs and novel drugs [56]. CCl_4_-intoxication causes critical liver damage in rats, which simulates acute hepatitis with symptoms similar to those seen in humans [57].

The induction of liver fibrosis by CCL_4_ was proven by histopathological examinations of liver tissues, which showed extremely disorganized hepatic architecture and marked thickening of the Glisson’s capsule due to fibrosis followed by degenerated hepatocytes with edema, as well as karyorrhexis nucleus and coagulative necrosis of major hepatocytes, and more eosinophilic cytoplasm with or without chromatin condensation and apoptosis. These histological changes are in line with other studies [58,59]. Furthermore, Masson’s trichrome stain confirmed the presence of an accumulation of mature collagen fibers (blue-stained), which indicated fibrotic lesions following intoxication by CCl_4_ [60]. In the same context, these histopathological lesions were associated with increased α-SMA-immunopositive HSCs in the hepatic lobules and in the area of these fibrotic lesions. Activated HSCs were identified by the appearance of α-SMA, when stimulated, HSCs become active and promote the synthesis and deposition of ECM, which progressively leads to the formation of fibrous scars in the compromised liver [61]. These changes in the liver architecture are associated with marked weight loss, hepatomegaly, and elevations in the serum bilirubin level, and activities of ALT, AST, and ALP, and a significant drop in albumin level. These results were in accord with numerous studies that confirm the fibrotic effects of CCl_4_ [62,63].

The CCl_4_-induced hepatocellular injury in the experimental animals model is mediated by oxidative stress [64,65]. The present data indicated a prominent elevation of MDA in liver tissue upon exposure to CCl_4_ indicating an onset of oxidative stress, which may terminally activate HSC causing hepatocyte degeneration and necrosis, leading to extracellular matrix deposition and the progression of fibrosis or cirrhosis. The oxidative stress in fibrosis rats was worsened by the impaired antioxidant systems as indicated by the decline in the hepatic contents of nuclear factor erythroid-2-related factor 2 (Nrf2), which is considered the master regulator of cellular redox homeostasis [66]. Several studies have found that stimulating Nrf2 significantly reduces liver fibrosis, indicating that Nrf2 is a promising target for the therapy of liver fibrosis [67,68].

All of these events activate the pathways of hepatic fibrogenesis including marked increase in the hepatic contents of TGF-β1, which plays a critical role in the genesis and development of chronic liver diseases [69,70]. Previous studies have reported increases in TGF-β1 levels in liver tissue and serum of CCl_4_-rats [69,71].

Increasing numbers of studies have demonstrated that many miRNAs family members play pro-fibrotic or anti-fibrotic roles by targeting genes collectively or individually by using certain signaling pathways to activate HSC and contribute to liver fibrosis [10]. However, the role of miRNAs and their specific mechanism of action in liver fibrogenesis still need to be clarified [72]. The observed histological and biochemical alterations in rats with fibrosis were accompanied by major molecular changes, including marked suppression of miR-22, miR-29c, and miR-219a and induction of their predicted target genes; TGFβR1, COL3A1, and TGFβR2, respectively.

These findings were consistent with earlier studies that indicated expression levels of miR-22 were downregulated in mouse fatty liver disease models [73]. TGFβR1 was identified as a potential target of miR-22 by computational analysis, where the strong negative correlation between the hepatic expression of miR-22 and TGFβR1 mRNA was confirmed in the current study by the pronounced upregulation of TGFβR1 expression in the fibrosis rat model (approximately 5.5-fold), which was consistent with a previous study [71,74], that found TGFβR1 expression to be increased in fibrotic livers of humans and rats in correlation with HSC proliferation and increased fibrosis. Our results suggest a clear regulatory influence of miR-22 on TGFβR1 mRNA, similar to that observed in the cardiac fibroblasts model, wherein the authors found that miR-22 can inhibit the expression of TGFβR1 at the posttranscriptional level [75]. Another study has shown that miR-22 suppresses proliferation and promotes the differentiation of C2C12 cells by targeting TGFβR1 in the myoblast proliferation model [15]. These findings highlight miR-22 as a potential protective and/or therapeutic target for liver fibrosis.

The downregulation of miR-29c has recently been associated with chronic liver inflammation and fibrogenesis in animal models and humans [13]. The documented lower hepatic expression of miR-29c was corroborated by previous research that showed downregulated expression of miR-29c in fibrotic rats [13,76].

The results presented in this study demonstrated that miR-29c expression was inversely correlated with the expression of the target gene COL3A1 as a potential target for miR-29c where COL3A1 expression was significantly upregulated in fibrotic liver compared to healthy liver tissue. COL3A1 induction can meet the requirements for induced drastic enlargement of liver fibrosis in rats because COL3A1 encodes collagen 1(III) chain, a precursor of collagen III [77], and serves as a ‘cell-binding’ of tissues.

In line with these data, the increased expression of COL3A1 after CCl_4_ administration of rats was reported [78]. In addition, Liu and colleagues performed a study on the role of COl3A1 mRNA expression in goose fatty liver, where target gene expression was increased in goose fatty liver with a strong inverse correlation with miR-29c [79]. Thus, miR-29c and its target may represent novel therapeutic strategies against hepatic fibrogenesis.

The results indicated the correlation between miR-219a expression and liver fibrosis, where miR-219a was distinctly decreased with advanced fibrosis, in which the expression of this level was negatively related to the fibrotic stage, indicating that miR-219a could be associated with the development and progression of liver fibrosis disease. Furthermore, TGFβR2 was identified to be a predicted target gene of miR-219a, with marked upregulation in CCl_4_-rats compared to control rats, and its expression was inversely correlated with miR-219, which could regulate the expression of TGFβR2 by directly binding to its 3′-UTR, whereas the TGF-β pathway contributes to the hepatotoxicity that influences the activation of the HSCs. Our results were consistent with previous studies that have indicated that TGFBR2 was a potential target of miR 219 [72]. Therefore, miR-219a is recommended as a promising therapeutic approach for liver fibrosis clinical intervention.

Despite substantial advances in understanding the pathophysiology of hepatic fibrosis, no antifibrotic medication is presently approved for use in humans. Several possible antifibrotic drugs and compounds, such as SIL, caffeine, and curcumin have shown antifibrotic properties but are not routinely used in clinical practice [80,81].

SIL is a flavonoid complex extracted from milk thistle seed with silibinin as the main constituent. Silibinin has many pharmacologic actions including antioxidant, anti-inflammatory, antifibrotic, and insulin resistance modulation [82]. Despite showing promise in animal models with improved liver function tests in humans, there is little evidence demonstrating its clinical benefit [83]. The crude SIL extract possesses no lipophilic properties and is poorly water soluble; thus, only about 20–50% is absorbed from the gastrointestinal tract after ingestion [84]. Overall, low bioavailability, poor stability, inefficient intestinal absorption, rapid excretion, multiple ring structure that impair simple diffusion, and elevated metabolism contribute significantly to reducing the concentration of SIL in the blood [85], resulting in a less therapeutic effect [86]. In the present study, we formulated and synthesized SCNPs in order to enhance the anti-fibrotic efficiency and bioavailability of SIL.

The treatment of the fibrotic rats with SIL formulated chitosan nanoparticle (SCNPs), SIL solution or nanocarrier, significantly improved the rate of weight gain, liver weight, and liver index % compared with untreated rats. The best effects were observed in the rats treated with SCNPs with completely normalized these parameters. The restoration of previous parameters may reflect the therapeutic effects of SIL and SCNPs against CCl_4_ intoxication, as the reverse of toxic influences and liver weight restoration relies on toxicants elimination and their metabolites from the liver [87].

At the histological and immunohistochemical (IHC) levels, the findings in this study suggest that SCNPs is potent than SIL and NPS alone, due to their enhanced solubility and bioavailability, which was supported through histopathology, and morphological analysis of the livers, where the animals treated with SCNPs showed a pronounced therapeutic effect against liver fibrosis with normal liver architecture comparable to the control group with negligible inflammatory infiltrate. The treatment with CNPs or SIL demonstrated moderate restorative influence on hepatic morphology. In addition, the results of Masson’s trichrome (MT) staining demonstrated the prominent effects of SCNPs in preventing collagen synthesis and deposition in the liver, where the liver sections from fibrotic rats treated with SCNPs had fewer fibers, while those treated with SIL and CNPs, respectively, had more fibers than SCNPs group. Thus, direct evaluation of extracellular matrix (ECM) deposition in hepatic tissue by MT staining clearly depicts the therapeutic efficiency of SCNPs against chronic hepatic fibrosis. Furthermore, immunohistochemical staining of α-SMA demonstrated a staining pattern in the SCNPs-treated group, similar to control animals, with sporadic α-SMA positivity by inhibition of HSCs activation with a more potent response than SIL and NPs alone. These effects were associated with considerable improvements in serum markers such as AST, ALT, ALP, bilirubin, and albumin. With SCNPs treatment, the majority of these parameters were entirely normalized.

SCNPs displayed better therapeutic effects than traditional drug SIL and the nanocarrier CNPs, which also showed a significant ameliorative effect on liver fibrosis. CNPs’ liver-targeting potential has been demonstrated and is related in particular to the size and surface properties of the polymer [88]. Because of its anti-tumor, antibacterial, antiulcer, immunostimulatory, and other special biological activities, chitosan has gained considerable interest as a medicinal material. The antioxidant function of chitosan has attracted the most attention recently [89]. All the treatment groups in this study improved the hepatic damage induced by CCl_4_ in rats through a significant decline in the MDA and increasing Nrf2 content in the hepatic tissue compared to the CCl_4_–intoxicated group. Animals treated with SCNPs demonstrated more potent suppression of CCl_4_-induced oxidative stress perhaps by increasing the Nrf2 content. These results suggested that the therapeutic effect of SIL-loaded NPs against liver fibrosis may be through high activation of the Nrf2 content to inhibit oxidative stress-mediated hepatocyte damage in the rat liver fibrosis model.

At the inflammatory level, the therapeutic effect of SIL, CNPs, and SCNPs on CCl_4_-induced liver fibrosis was consistent with their ability to reduce the hepatic TGF-β1 production with the best effect in the rats treated with SCNPs. Previous studies have indicated that the incorporation of SIL with CNPs ameliorates the hepatic damage through downregulating the liver stellate cells and attenuation of Kupffer cells [50].

At the molecular level, SIL, CNPs, and SCNPs significantly ameliorated the hepatic expression of miR-22, miR29c, and miR-219a after CCl_4_-induced liver fibrosis in rats and even became significantly higher than the control values. Conversely, the increased expression of miR-22, miR29c, and miR-219a was associated with downregulation of the expression of the target genes TGFβR1, COL3A1, and TGFβR2, respectively. Nevertheless, SCNPs demonstrated a more superior response to downregulation of genes implicated in hepatic fibrosis than SIL and NPs alone. Thus, the observed boosting effect on of hepatic expression of miRNAs may be regarded as the pivotal event in the therapeutic effects of SCNPs against CCl_4_-induced liver damage in rats.

The superior effects of SCNPs over the Sil solution can be explained by the fact that the nano-formulations are better than the usual low-molecular-weight drugs in many ways. The used CNPs as a nano-carrier protects the SIL, enhancing its solubility and bioavailability, reducing liver degradation and renal secretion, all of which lead to extended circulation time. Furthermore, the biological effects of the nano-carrier itself as the CNPs alone have many hepatoprotective and therapeutic effects [50].

Several studies have shown that SIL prevents the development of liver fibrosis in animal fibrosis largely due to its anti-fibrogenic effects at the molecular level. Previous studies have demonstrated improved protection influence of SIL on rat liver damage caused by thioacetamide in rats by increased levels of miR-122, miR-192, and miR-194 levels, which contributed to ameliorating liver damage [90]. Furthermore, SIL reduced profibrotic gene expression in the liver of obese diabetic Otsuka Long-Evans Tokushima rats [91]. A study led by Meng revealed that SIL ameliorates myocardium fibrosis in rats with diabetic cardiomyopathy by inhibiting TGF-β1/Smad signaling, which is involved in TGFβR1 and TGFβR2 expression activation [92]. Conversely, previous studies have reported that chitosan inhibits the growth of fibroblasts in the Achilles tendon of rats, which is improved by the high expression of miR-29b and its downregulation of TGF-β1 [93].

The epigenetic effects of SIL or CNPs were reported previously. Silibinin has been shown to have anti-prostate carcinoma through epigenetic mechanism by inducing the activity of total DNA methyltransferase and a decrease in the expression of histone deacetylases 1–2. In addition, CNPs have anti-metastatic activity in esophageal cancer-associated fibroblasts through modulating gene expression [94]. In the same context, according to a study on the epigenetic effect of CNPs on global DNA methylation conducted by Sooklert and colleagues, CNPs induced global DNA hypomethylation in keratinocyte cells [95].

From the above discussion, we assume that the SCNPs formulation has more potent anti-fibrotic efficiency than the SIL drug, and the main effects may be mediated through (1) induction of hepatic miRNAs (miR-22, miR-29c, and miR-219a) resulting in the inhibition of expression of their targets, including TGFβR1, COL3A1, and TGFβR2; respectively; (2) anti-inflammatory effects; (3) enhancing antioxidant activities; and (4) inhibiting fibrogenesis and boosting the structural and functional integrity of hepatocytes. The study’s main limitation is that the direct epigenetic mechanism(s) of SIL, CNPs, and SCNPs require further investigation. Furthermore, further investigations are required to explore the pharmacokinetics and pharmacodynamics properties of SCNPs and to confirm their direct molecular targets.

## 4. Materials and Methods

### 4.1. Chemicals and Regents

Silymarin powder was generously gifted by the Egyptian Group for Pharmaceutical Industries, (Cairo, Egypt). Chitosan (deacetylation 93%, MW 161.16 (kDa) was purchased from Oxford Lab Chem (Maharashtra, India), Sodium Tripolyphosphate, MW 367.86 was procured from Alpha Chemika (Mumbai, India). All other chemicals and reagents used throughout the experiments were of the highest analytical grade available.

### 4.2. Preparation and Characterization of Nanoparticles

#### 4.2.1. Preparation of Chitosan Nanoparticles

According to a study carried out by Calvoet et al. and Gupta et al. [54,96], the preparation of chitosan nanoparticles (CNPs) was carried out using the ionotropic gelation method. Chitosan undergoes ionic gelation and precipitates to form spherical particles as a result of the complexation of opposing charges (positive/negative) [97]. In brief, low molecular weight chitosan was dissolved in the aqueous solution of acetic acid to form a 2 mg/mL chitosan solution (a total of 100 mL was prepared; thus, 200 mg of chitosan was dissolved in 100 mL of 2% acetic acid). The pH of this solution was adjusted to 5.0–5.5 pH by adding an aqueous NaOH solution [98]. The solution was stirred for ~40 min. Then, TPP (as a crosslinker) was dissolved in water (10 mg of TPP in 20 mL of ultrapure water) to make a concentration of 0.5 mg/mL [33], and the pH was altered to 2.0 using HCl [99]. At room temperature, 20 mL of TPP was slowly dropped into the chitosan solution while vigorously magnetically stirring. The reaction was carried out for ~45 min, continuously stirred, and eventually the resulting suspension. The suspension was then centrifuged in order to separate unreacted chitosan and TPP from nanoparticles. The suspension was centrifuged at 11,000 rpm at 4 °C for 30 min. The supernatant was discarded, and the pellet was resuspended in 10 mL of Milli-Q-water till the complete dissolution of the pellets. Centrifugate steps were repeated 2 times (total time was 3 rounds). After the 3rd round, each tube was resuspended in 15 mL of Milli-Q-water and then sonicated for 30 min using a bath sonicator; Branson 1510r-Mth, (Watertown, MA, USA) and stored at 4 °C. For the measurement of FTIR, the part of the prepared NPs was dried by the lyophilizer.

#### 4.2.2. Synthesis of Silymarin–Chitosan Nanoparticles

Silymarin-loaded chitosan nanoparticles (SCNPs) were synthesized by the ionotropic gelation method as described by Gupta et al. and Nagpal [54,100]. In this method, positively charged chitosan interacted with negatively charged groups of polyanions. In this experiment, LMW chitosan was dissolved in the aqueous solution of acetic acid to form a 2 mg/mL chitosan solution (a total of 100 mL was prepared; thus, 200 mg of chitosan was dissolved in 100 mL of 2% acetic acid). The pH of this chitosan solution was adjusted to 5 using a 1.0 M NaOH solution. The solution was stirred for ~40 min. The drug solution was prepared by dissolving silymarin (SIL) (10 mg) in 1 mL of ethanol, sonicated for 2 min, and this solution was added dropwise slowly into the solution of chitosan, followed by a 20 mL dropwise addition of TPP solution. The pH of this solution was adjusted to 5 using 0.1 M HCL with constant stirring for 3 h at 500 rpm using a magnetic stirrer. The nanoparticle suspension was centrifuged at 15,000 rpm at 10 °C for 30 min. The supernatant was removed for analysis of free silymarin, and the pellets were washed twice before being redispersed in a constant volume of 10 mL of Milli-Q^®^ water (Medical Nanotechnology Laboratory, Center of Excellence, Alexandria University, Alexandria, Egypt) and sonicated for 20 min. Three milliliters of this suspension was further diluted 10 times, sonicated for 10 min, and evaluated for some characterizations such as particle size, size distribution, and zeta potential. The undiluted nanoparticles were lyophilized using a lyophilizer (Alpha 2–4 LD plus CHRIST, Germany) after adding d-mannitol as a cryoprotectant in order to avoid particle agglomeration.

### 4.3. Physicochemical Characterizations of Nanoparticles

#### 4.3.1. Dynamic Light Scattering and Zeta Potential Analysis

The Dynamic light scattering (DLS) was used to determine the particle size (hydrodynamic diameter), and polydispersity index (PDI), and ZP measurements of CNPs and drug-loaded SCNPs were performed three times for each sample at 25 °C on a Zetasizer Nano ZS (Nano and zeta Sizer Malvern, Grove, UK), with a backscattering detection angle of 173°. Before the analysis, the nanoparticle samples were diluted with ultrapure distilled water and sonicated.

#### 4.3.2. Transmission Electron Microscopy Analysis

The size and morphological features of CNPs and SCNPs were studied by transmission electron microscopy (TEM). A few drops of diluted CNPs or SCNPs suspensions were placed on a formvar carbon-coated 200 mesh copper TEM grid, negatively stained with 1% uranyl acetate and lead acetate (*w*/*v*) for 10 min, and allowed to dry. The grid was placed in the specimen holder, and the instrument was adjusted. The TEM images of NPs were taken using transmission electron microscopy–JEOL–JSM–1400 PLUS (Peabody, MA, USA).

#### 4.3.3. Fourier Transforms Infrared Spectroscopy Study

FT-IR spectroscopy was used to analyze the functional groups on SIL powder as control, CNPs, and SCNPs by the potassium bromide (KBr) pellet method. First: the solutions of SIL, CNPs, or SCNPs were lyophilized, and KBr was added to an agate mortar and ground to a fine powder. Then, the dried powders were mixed separately with KBr powder at a ratio of 1:10. The mixtures were ground for 2–4 min and pressed by a hydraulic press. The mixed powders were then pressed for 1–2 min to form pellets. The pellets were cautiously transported to the FTIR sample holder. The measurements were carried out in the range between 400 and 4000 cm^−1^ using the Vector22 FTIR Spectrometer (Bruker, Germany).

#### 4.3.4. Drug Entrapment Efficiency and Loading Capacity

The trapped drugs inside the nanoparticles as a percentage of total drugs are referred to as entrapment efficiency (EE). Briefly, 10 mL of SCNPs suspension were separated from their aqueous medium containing free SIL by centrifugation at 15,000 rpm, 4 °C, for 30 min. Then, the sample was redispersed in ultra-pure water. The concentration of SIL in the supernatant was diluted and analyzed by a UV spectrophotometer at 285 nm. A calibration curve of standard SIL concentrations vs. absorbances was constructed. The EE % and drug loading capacity (LC %) of SIL in SCNPs were calculated using the following equations:

$$\mathrm{EE}\;\%=\frac{\left(\mathrm{Amount}\;\mathrm{of}\;\mathrm{SIL}\;\mathrm{added}\;-\;\mathrm{free}\;\mathrm{SIL}\;\mathrm{in}\;\mathrm{supernatant}\right)}{\;\mathrm{Amount}\;\mathrm{of}\;\mathrm{SIL}\;\mathrm{added}}\times 100\;\mathrm{LC}\;\%=\frac{\left(\mathrm{Amount}\;\mathrm{of}\;\mathrm{SIL}\;\mathrm{added}\;-\;\mathrm{free}\;\mathrm{SIL}\;\mathrm{in}\;\mathrm{supernatant}\right)}{\;\mathrm{Nanoparticles}\;\mathrm{weight}}\times 100$$

#### 4.3.5. In Vitro Drug Release Study

The in vitro release of SIL from SCNPs was evaluated for a period of 48 h by the method adopted by Radu et al. [48]. The dosage form of SCNPs was inserted into the release media, which is kept at a constant temperature, after which the release of drugs is evaluated by sampling and detection of SIL in the release media. Briefly, 10 mg of SCNPs sample dry powder was dispersed in 10 mL of PBS containing 0.1% tween 20. Then, it was incubated in a shaking water bath at 37 °C at 250 rpm. At the time intervals 0.5, 1, 2, 4, 8, 12, 16, 20, 24, 28, 32, and 36 h, 5 mL of supernatant was withdrawn and replaced by 5 mL of fresh dissolution medium at 37 ± 0.5 °C to maintain the overall volume constant until the end of the experimental period to examine the release kinetics of the SIL drug. The SIL released in these fractions was evaluated by measuring the absorbance of solutions at 285 nm. The SIL release data were adjusted by converting the drug concentration into a percentage of the cumulative SIL release using the following formula [101]:

$$\;\%\;\mathrm{of}\;\mathrm{cumulative}\;\mathrm{drug}\;\mathrm{release}=\frac{\;\mathrm{Released}\;\mathrm{SIL}\;\mathrm{from}\;\mathrm{NPs}\;\mathrm{at}\;\mathrm{time}}{\;\mathrm{Amount}\;\mathrm{of}\;\mathrm{SIL}\;\mathrm{added}\;\mathrm{in}\;\mathrm{NPs}}\times 100$$

### 4.4. In Vivo Study

#### 4.4.1. Animals

A total of thirty pathogen-free male Sprague–Dawley (SD) rats and weighed approximately 250 ± 10 g at 3–4 months of age were used. The animals were purchased from the animal house facility of the Medical Technology Center (MTC), Medical Research Institute (MRI), Alexandria, Egypt. Animals were housed in individually ventilated cages under controlled conditions (22 °C, and 12:12 hour’s light/dark cycle). They had free access to food and water, and constant environmental conditions prior to experimentation and thereafter. All animals were acclimatized in the laboratory for two weeks prior to the start of the experiments.

#### 4.4.2. Ethical Statement

All procedures were performed in accordance with the Institutional Animal Care and Use Committee (IACUC)-Alexandria University, Egypt (Approval No: AU0122132423). The study also follows ARRIVE guidelines and complies with the National Research Council’s guide for the care and use of laboratory animals.

#### 4.4.3. Establishment of Liver Fibrosis Model

For 10 weeks, rats were injected intraperitoneal (i.p.) with carbon tetrachloride (CCl_4_) (1 mL/kg) dissolved in olive oil (1:1 *v*/*v*) [102], three times weekly, and then two times weekly for two weeks to induce liver fibrosis.

#### 4.4.4. Experimental Design

The animals were separated into five groups (6 rats each):the control group: the rats received ip injection of olive oil; fibrosis group was induced as previously described and received no treatment; SIL-treated group: rats with liver fibrosis were treated with SIL solution at a dose of 100 mg/kg in olive oil [103]; CNPs-treated group: rats with liver fibrosis were treated with CNPs at a dose of 50 mg/kg; SCNPs-treated group: rats with liver fibrosis were treated with SCNPs at a dose of 50 mg/kg [54]. All treatments were administered by oral gavage for 30 days.

#### 4.4.5. Sample Collection and Tissue Preparation

At the end of the treatment period, all animals were overnight fasted, weighed, and anesthetized by deep isoflurane inhalation. The blood samples were obtained by cardiac puncture and centrifuged at 3000 rpm at 4 °C for 20 min to separate sera for the biochemical analysis. The livers were dissected out, washed, and weighted to calculate the liver index according to the following:

$$\mathrm{Liver}\;\mathrm{Index}\;\mathrm{Calculation}=\left[\;\mathrm{Liver}\;\mathrm{weight}\;\left(g\right)/\mathrm{Body}\;\mathrm{weight}\;\left(g\right)\right]\times 100$$

The liver tissues were divided into three aliquots; the first was processed in 10% normal formalin for H&E staining, Masson’s trichrome staining (MT), and immunohistochemistry (IHC). The second aliquot was quickly frozen and stored at −80 °C to be used for total RNA extraction to assess gene expression using quantitative real-time polymerase chain reaction (qRT-PCR) analysis. The remaining aliquot was taken for homogenization in cold 0.1 mM PBS in a ratio (1:9) to be used for ELISA and lipid peroxidation measurements.

### 4.5. Methods

#### 4.5.1. Serum Biomarkers for Liver Function Tests

Aspartate aminotransferase (AST), alanine aminotransferase (ALT), and alkaline phosphatase (ALP) activities, as well as total bilirubin and albumin levels, were measured using commercially available kits (Biosystems S.A. Costa Brava 30, Barcelona, Spain) in accordance with the manufacturer’s instructions.

#### 4.5.2. Malondialdehyde (MDA) as Index of Lipid Peroxidation

MDA in homogenate was assayed as thiobarbituric acid reactive substances (TBARS) according to the method of Draper and Hadley [104]. The sample was heated with thiobarbituric acid (TBA) at low pH (3.5). The absorbance of the resulting pink chromogen was assayed at 532 nm.

#### 4.5.3. ELISA Measurements

The liver concentrations of TGF-β1 and Nrf2 in the tissue supernatants were assayed using rat specific ELISA kits (Chongqing Biospes Co., Chongqing, China) according to the manufacturer’s instructions.

#### 4.5.4. Gene Expression Analysis

The RNA content of hepatic tissues was obtained using miRNeasy mini kit (Qiagen, Hilden, Germany) according to the manufacturer’s directions. The concentration of extracted RNA was determined using nanodrop (Jenway™ Genova Nano micro-volume Spectrophotometer, Staffordshire, UK). The total RNA was reverse transcribed using miScript II RT Kit (Qiagen, Germany) according to the manufacturer’s recommendations. The reverse transcription reaction was carried out using the miScript II RT Kit, and HiFlex Buffer was utilized to promote the conversion of all RNA species into cDNA.

##### Assessment of Hepatic MicroRNAs (miRNA-22, miRNA-29c, and miRNA-219a)

The obtained cDNA was used for qPCR analysis of mature miRNAs (miRNA-22, miRNA-29c, and miRNA-219a) using Primer Assays (forward primers) and the miScript SYBR Green PCR Kit (Qiagen, Germany), which contains the Universal Primer (reverse primer) and QuantiTect SYBR Green PCR Master Mix (Qiagen, Germany). Control U6 (Qiagen, Germany) was used as a reference normalizing gene for miRNAs quantification. The PCR amplification conditions started with an initial denaturation for 10 min at 95 °C and then amplification by 40 cycles as follows: denaturation at 95 °C for 15 s, annealing at 50 °C for 20 s, and extension at 60 °C for 20 s. Data were collected and analyzed using Rotor-Gene Q-Pure Detection version 2.1.0 (build 9) (Qiagen, Germantown, MD, USA).

##### Assessment of Hepatic Expression of TGFβR1, TGFβR2, and COL3A1 Genes

The obtained cDNA was used to assess the hepatic expression of TGFβR1, TGFβR2, and COL3A1 genes by PCR using QuantiTect SYBR Green PCR Master Mix (Qiagen, Germany). The control GAPDH (Qiagen, Germany) was used as a reference normalizing gene for mRNAs quantification. The primer sets used for PCR amplification are presented in Table 2. The PCR amplification conditions started with an initial denaturation for 5 min at 95 °C and then amplification by 40 cycles as follows: denaturation at 94 °C for 15 s, annealing at 55 °C for 20 s, and extension at 60 °C for 40 s.

##### Relative Quantification of Gene Expression

The relative expressions of both miRNAs or mRNAs were quantified relative to the expression of the reference genes (U6 for miRNAs, and GAPDH, for mRNAs) in the same sample by normalizing the threshold cycle (Ct) values of target miRNAs or mRNAs to that of U6 or GAPDH, respectively, using the ΔΔCt method. The results were expressed as relative expression ratio or fold-change compared with the ‘Control group’ according to the Livak method [105].

### 4.6. Histopathology Study

The liver tissues were quickly collected and treated in 10% neutral-buffered formalin for 24 h, after which they were dehydrated in gradually increasing concentrations of ethanol (from 70% to 100%), cleared in xylene, and embedded in paraffin. A microtome was used to cut five-micron thick paraffin slices (Leica RM 2155, London, UK). For histopathology, the sections were stained with hematoxylin and eosin (H&E) [106]. Masson’s trichrome (MT) staining was used to detect collagen fibers in the fibrous lesion sites, and the collagen accumulation of every group was computed using ImageJ software [107]. All section photos were taken using a Leica^®^ microscope (wetzlar, Germany), in conjunction with an Am Scope^®^ microscope digital camera. ImageJ software was used to perform a quantitative study of liver fibrosis (as a percentage) on sections stained with MT stain (Image J 1.47v, National Institute of Health, Bethesda, MD, USA). The colors settings in the ImageJ software were kept in sync with the blue-stained area measurements in the samples at all times; these measurements were taken for images with at least 10 different fields per section at a magnification power of ×100 [107]. The following criteria were used to calculate fibrosis stage scores: score (0) absent fibrosis; score (1) slight fibrosis; score (2) mild fibrosis; score (3) moderate fibrosis; score (4) severe fibrosis. The assessment was previously described [108].

### 4.7. Immunohistochemistry (IHC) Study

Immunohistochemical detection of alpha-smooth muscle Actin (α-SMA) as a marker for hepatic stellate cells (HSCs) activation was performed using Avidin-Biotin-peroxidase Complex (ABC) Methods described by Hsu et al. [109]. Briefly, 3 µ thick paraffin sections were deparaffinated in xylene and rehydrated in a graded series of ethanol. Then, the tissue sections were incubated in metanol-0.3% H_2_O_2_ at room temperature for 30 min. The sections were washed in distilled water for 5 min, followed by PBS at pH 7.3 for 5 min [110], and then blocking for non-specific binding sites was performed with 5% Bovine Serum Albumin (BSA) in PBS for 1 h at room temperature. The sections were incubated with Anti-α-SMA primary antibody (Abcam, Cambridge, UK) overnight at 4 °C at a concentration of 1 g/mL containing 5% BSA in PBS. The slides were washed 3 times by PBS at pH 7.4, and then were incubated with Goat Anti-Rabbit IgG H&L (HRP) secondary antibody for 1 h at room temperature. The sections were washed by PBS at pH 7.4 for 3 times and incubated for 10 min in 0.02% Diaminobenzidine (DAB) containing 0.01% hydrogen peroxide. Finally, sections were counterstained by hematoxylin, and the slides were visualized under a microscope. Immunohistochemical reactions positive percentages (average number of positive cells per 10 field’s high power ×400) were counted by using ImageJ software (Image J 1.47v, National Institute of Health, Bethesda, MD, USA).

### 4.8. Statistical Analysis

The data were entered into a computer and analyzed using the IBM SPSS version 20.0 software program. (Armonk, NY, USA: IBM Corporation). To confirm that the distribution was normal, the Kolmogorov–Smirnov test was used. The mean and standard deviations were used to describe quantitative data. The significance of the data obtained was determined at the 5% level. The F test (ANOVA) was performed for pairwise comparisons, followed by the Tukey post hoc test.

## 5. Conclusions

In the present study, we successfully prepared silymarin-loaded chitosan nanoparticles (SCNPs) using the ionotropic gelation technique. The formulated nanoparticles showed optimized physicochemical properties for efficient delivery of encapsulated silymarin to target liver cells. In vitro drug release of SCNPs showed higher drug release and bioavailability compared with CNPs. Furthermore, in the in vivo study, the results revealed that the obtained SCNPs showed greater effects against CCl_4_ toxicity-induced liver fibrosis with the diminished oxidative damage and increased antioxidant defense system compared with SIL itself. This might occur via targeting and boosting of the hepatic expression of protective microRNAs; miR-22, miR-29c, and miR-219a, resulting in downregulation of the major mediators of fibrosis. These effects, concurrently with the reduction of oxidative stress, lowering of TGFβ-1, and stimulation of Nrf2, led to the blocking of pathogenic pathways involved in the development of liver fibrosis, chronic inflammation, and collagen deposition. These results suggest that the nanoformulation of SIL with CNPs is a viable option for its oral delivery as a potential therapeutic entity in the treatment of liver fibrosis.

## Figures and Tables

**Table 1:** Effects of SIL, CNPs, and SCNPs on serum liver function parameters.

Groups	Parameters				
	AST (U/L)	ALT (U/L)	ALP (U/L)	Total Bilirubin (mg/dL)	Albumin (g/dL)
Control	4.13 ^a^ ± 0.21	0.41 ^c^ ± 0.10	103.5 ^d^ ± 13.79	44.34 ^d^ ± 7.42	112.6 ^d^ ± 12.44
Liver fibrosis	3.12 ^c^ ± 0.13	1.16 ^a^ ± 0.30	253.3 ^a^ ± 19.62	131.7 ^a^ ± 13.70	196.4 ^a^ ± 17.76
Liver fibrosis + SIL	3.48 ^b,c^ ± 0.24	0.74 ^b^ ± 0.21	147.9 ^c^ ± 16.72	62.19 ^c^ ± 9.02	133.9 ^b–d^ ± 12.75
Liver fibrosis + CNPs	3.30 ^b,c^ ± 0.21	0.83 ^b^ ± 0.12	152.0 ^c^ ± 14.06	68.67 ^c^ ± 5.79	149.8 ^b,c^ ± 19.07
Liver fibrosis + SCNPs	3.62 ^b^ ± 0.08	0.61 ^b,c^ ± 0.10	137.7 ^c^ ± 14.54	53.3 ^c,d^ ± 6.62	129.7 ^c,d^ ± 19.87

Data are presented as mean ± SD (*n* = 6). The means of the groups in the same column with the same superscript letter are not significantly different while the means with different letters are significantly differed according to the ANOVA test, followed by the post hoc test (Tukey). At *p* ≤ 0.05, the difference was statistically significant. Abbreviations: AST, aspartate transferase; ALT, alanine transaminase; ALP, alkaline phosphatase.

**Table 2:** The primer sequences for target genes used in the study.

Gene	Sequence	
COL3A1	Forward	5′-AAC GGA GCT CCT GGC CCC AT-3′
	Reverse	5′-ATT GCC TCG AGC ACC TGC GG-3′
TGFβR1	Forward	5′-GCT GAC ATC TAT GCA ATG GG-3′
	Reverse	5′-ATA TTT GGC CTT AAC TTC TGT TC-3′
TGFβR2	Forward	5′-CCA GGG CAT CCA GAT CGT GTG-3′
	Reverse	5′-TAG TGT TCA GGG AGC CGT CTT-3′
GAPDH	Forward	5′-GGG TGT GAA CCA CGA GAA ATA-3′
	Reverse	5′-AGT TGT CAT GGA TGA CCT T-3′

## Data Availability

Data will be available by request from the corresponding authors.
